# Evaluation of a pilot of nurse practitioner led, GP supported rural palliative care provision

**DOI:** 10.1186/s12904-016-0163-y

**Published:** 2016-11-09

**Authors:** Geoffrey Keith Mitchell, Hugh Edgar Senior, Michael Peter Bibo, Blessing Makoni, Sharleen Nicole Young, John Patrick Rosenberg, Patsy Yates

**Affiliations:** 1 Discipline of General Practice, University of Queensland School of Medicine, Herston Rd, Herston, 4006 Queensland Australia; 2 College of Health, Massey University, Auckland, New Zealand; 3 Southern Cross Care, Ipswich, Australia; 4 West Moreton Hospital and Health Service, Ipswich, Australia; 5 School of Nursing, Queensland University of Technology, Brisbane, Australia

**Keywords:** Palliative care, Primary care, Nurse practitioner, General practice, Organisation of care, Case conferences

## Abstract

**Background:**

Providing end of life care in rural areas is challenging. We evaluated in a pilot whether nurse practitioner (NP)-led care, including clinical care plans negotiated with involved health professionals including the general practitioner(GP), ± patient and/or carer, through a single multidisciplinary case conference (SMCC), could influence patient and health system outcomes.

**Methods:**

Setting – Australian rural district 50 kilometers from the nearest specialist palliative care service. Participants: Adults nearing the end of life from any cause, life expectancy several months. Intervention- NP led assessment, then SMCC as soon as possible after referral. A clinical care plan recorded management plans for current and anticipated problems and who was responsible for each action. Eligible patients had baseline, 1 and 3 month patient-reported assessment of function, quality of life, depression and carer stress, and a clinical record audit. Interviews with key service providers assessed the utility and feasibility of the service.

**Results:**

Sixty-two patients were referred to the service, forty from the specialist service. Many patients required immediate treatment, prior to both the planned baseline assessment and the planned SMCC (therefore ineligible for enrollment). Only six patients were assessed per protocol, so we amended the protocol. There were 23 case conferences. Reasons for not conducting the case conference included the patient approaching death, or assessed as not having immediate problems. Pain (25 %) and depression (23 %) were the most common symptoms discussed in the case conferences. Ten new advance care plans were initiated, with most patients already having one. The NP or RN made 101 follow-up visits, 169 phone calls, and made 17 referrals to other health professionals. The NP prescribed 24 new medications and altered the dose in nine. There were 14 hospitalisations in the time frame of the project. Participants were satisfied with the service, but the service cost exceeded income from national health insurance alone.

**Conclusions:**

NP-coordinated, GP supported care resulted in prompt initiation of treatment, good follow up, and a care plan where all professionals had named responsibilities. NP coordinated palliative care appears to enable more integrated care and may be effective in reducing hospitalisations.

## Background

The population is ageing rapidly. In Australia, by 2026 the proportion of people over 65 will increase from the current 13 to 23 %, with 5 % being over 85 years old [1]. With this will come more people with a range of conditions threatening life, and the number of actual deaths will increase rapidly. The health service impact of this trend is apparent now, with the annual number of specialist palliative care admissions in Australia increasing by over 50 % in the decade to 2012–13 [2]. A re-evaluation of the health system is needed to ensure sustainable care. This is particularly the case in rural areas where the ratio of palliative medicine specialists to population is low and alternative means of service delivery are required. The role of community and primary care services is especially important in rural settings, as are models that involve health professionals with extended scope of practice, such as Nurse Practitioners (NP).

In Australia, the health system is based on general practices being the first point of care. Specialist services and community based nursing and allied health support is usually accessed through referral from general practitioners (GPs). Virtually all Australians have access to a GP: over 80 % see a GP every year [3]. NPs in Australia are registered nurses who are educated and endorsed to function autonomously and collaboratively in an advanced and extended clinical role. The NP role includes assessment and management using nursing knowledge and skills. The role may include, but is not limited to, the direct referral of patients to other healthcare professionals, prescribing medications and ordering diagnostic investigations [4]. NPs work independently within an agreed scope of practice, usually in collaboration with the patient’s treating team including the patient’s GP. NPs as primary care providers are uncommon, except in remote areas with limited availability of GPs. They are much more commonly employed within service providing organisations. Both GPs and to a more limited extent NPs can claim fees from Australia’s universal health insurance scheme (Medicare). However, health services are funded and administered by different levels of government, and coordination of services can be problematic.

**Fig. 1:**
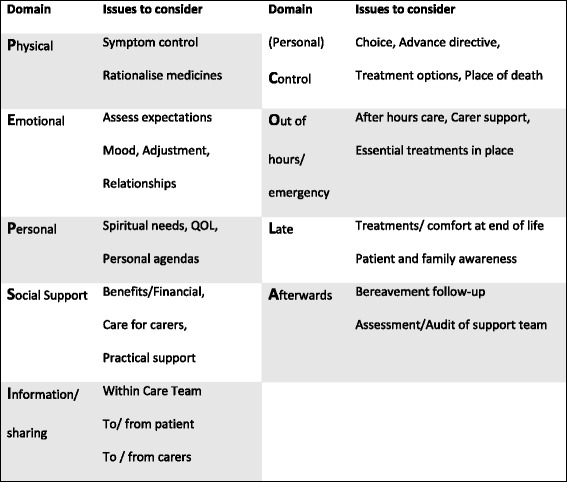
The Gold Standards framework for care plan development [5]

The combination of fragmented funding and administrative arrangements, and a small health professional workforce in rural settings can lead to suboptimal health care. When the complexity of care needs is significant, such as at the end of life, health care delivery for rural patients can be compromised. To reduce the fragmentation of service delivery and support the role of GPs in end of life care, we previously piloted the use of single multidisciplinary case conferences (SMCCs) in the context of a large regional city. The SMCC is a semi-structured case review following the PEPSI COLA process (Fig. 1) described by the Gold Standards Framework [5, 6] in the UK. This ensures that the patient’s and carer’s needs are examined comprehensively, and a care plan outlining future integrated care is developed. The participants in the pilot SMCCs were a specialist community based clinical nurse caring for the patient, the patient’s GP, and a palliative medicine specialist who acted as moderator and wrote the report. The patients had end stage heart and/or lung disease. The output of the case conference is a negotiated clinical care plan which describes current and anticipated patient and carer needs, proposes treatment plans, and specifies which clinician is responsible for which action. Through a before and after design, we demonstrated a probable major impact on health service utilisation in those disease groups, with statistically significant, large reductions in hospitalisations, emergency department visits, and length of stay after the case conference [7], and per patient savings of around $AU49,000 [8]. This confirms previous RCTs involving more than 600 participants of single case conferences in mainly cancer populations that demonstrated reduced service utilization [9], better maintenance of functional capacity [9] and improved quality of life [10]. Qualitative work demonstrates that single case conferences are feasible and acceptable [11, 12].

Given the unique challenges faced by patients outside of metropolitan settings, we wanted to determine the extent to which case conferencing could be applied in non-metropolitan settings using non-medical specialists in the consulting role. In 2014, we established a partnership with a regional primary health care network to develop and pilot a suitable model of care provision. This network had completed a comprehensive needs assessment which identified that there was a high, and unmet, demand for after-hours medical care experienced by patients at the end of life [13], particularly in rural areas of the region. The review also identified a severe shortage of qualified palliative medicine specialists in the district. Given the limited medical workforce in this setting, a decision was made to evaluate the extent to which a palliative care-focused NP who provided a coordinating, advisory and treatment role in assessment and care planning for people at end of life could improve access to care and health outcomes.

Therefore, the aim of this project was to pilot a NP-coordinated care planning project, targeting people living in a rural area nearing the end of their lives, and principally with non-malignant disease. The same format to that used in our pilot [7] was proposed, although specialist input was provided from the palliative care endorsed NP rather than a palliative medicine specialist. We sought to demonstrate positive patient and carer outcomes and a reduction in after-hours service requirements in the target population by identifying and addressing patient and carer needs proactively.

The objectives of the project were to assess the impact of this form of service provision on:

- Patient and carer outcomes
- Health care utilization
- Provider experience

## Methods

### Setting

We conducted a pilot of a NP-led, GP supported care provision, based on the previously published study of GP-specialist SMCCs in life-limiting heart and/or lung disease in the West Moreton Region of Queensland, Australia [7]. Funding was available for a 9-month evaluation, which effectively restricted recruitment into the study to a 6 month period. Southern Cross Care (SCC), a provider of aged care services in the district, was contracted to implement the palliative care NP coordinated service.

**Fig. 2:**
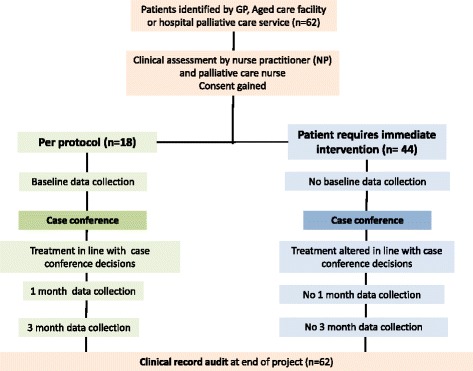
Study flow chart

We aimed to recruit 30 patients with frailty, organ failure or cancer from the area’s general practitioners and residential aged care facilities (RACF), to provide sufficient breadth of clinical and organizational settings to assess the model. We proposed a comprehensive evaluation of health service utilization and patient and carer outcomes, following the methods of the original project [7] After 2 months and slow recruitment, we expanded the recruitment pool to include patients discharged from the local specialist palliative care unit, in an attempt to reach our recruitment target. Many, but not all, of the patients referred from the palliative care unit were far more ill than those for whom the original project and evaluation had been planned, and had clinical problems requiring urgent attention. Hence treatment had to be commenced as soon as they were identified, before the planned baseline data could be acquired, and before the case conference was held (Fig. 2). These patients did not meet the project’s inclusion criteria, and so could not be included in the planned evaluation. However not allowing access to the new service was unethical, so we decided to conduct a per protocol assessment and a second evaluation of the entire cohort, documenting their demographics and the nature of the care provided by the NP-led model of care provision.

### Per protocol assessment

#### Eligibility criteria

Eligible patients were identified by the patient’s referring practitioner as having an advanced disease or frailty, and being at risk of dying within the next 12 months as determined by a “no” answer to the Surprise Question (SQ): “Would I be surprised if this person were to die in the next 12 months?” (SQ+) [14]. The patient’s GP had to agree to participate in a case conference. Patients at high risk of death within a month, unable to provide informed consent or unable to comprehend spoken English were not eligible. The patient’s carer was asked to provide consent for data collection, but non-consenting of the carer did not exclude patient participation.

#### Process

Patients were identified by the GP or SCC staff as being SQ+. The SCC team, comprising the NP and a registered nurse (RN) conducted a pre-case conference clinical assessment at the patient’s home, and gained informed consent for the research study from the patient and caregiver (if present). Informed consent was obtained from all health professionals including the patient’s GP.

We aimed to hold a SMCC between the patient’s GP, the NP and at least one other health professional involved in the patient’s care. (the minimum requirement for receiving funding from Medicare). If they wished, patients and/or their carers could participate. SMCCs were either face-to-face or by videoconferencing. The SMCC followed the Gold Standard Framework’s PEPSI-COLA assessment structure [6] leading to the development of a comprehensive care plan and allocation of responsibility for each task (Fig. 1). Emphasis was placed on facilitating communication between the GP and NP and other health professionals, after-hours emergency plans, educating the primary carer on how to deal with common anticipated problems, and systematically addressing the needs and concerns of primary carers using a Carer Needs Checklist [15]. The plan was completed by the NP and distributed for implementation by all health professionals involved.

Patients and carers were interviewed at home at baseline (before the conduct of the case conference and enactment of the plan), 1 and 3 months by a research assistant not involved in care provision. Data collection comprised a series of validated questionnaires (see Outcome Measures), and questions related to experience of the health service, administered by a research assistant at home, after consent was gained by the nursing team. In addition, formal qualitative interviews were conducted with patients and carers at baseline and 1 month to obtain more detailed information about the processes and experience of participating in the SMCC. Patients and carers were interviewed separately. The findings from this qualitative component will be published separately.

#### Outcome measures

Patient-reported outcomes were assessed using a range of measures. Depression and Anxiety were assessed using the Hospital Anxiety and Depression Scale (HADS)) [16]. This scale obtains seven scores rated from 0 (none) to 3(most severe) for each of anxiety and depression. If the total score for each of anxiety and depression is ≤7, the condition is considered normal; 8–10 borderline and ≥11 severe. Palliative symptoms were assessed using the Palliative Outcomes Scale (POS)) [17]. This scale includes ten questions measuring patient perceived palliative symptom burden in the last three days. Each question is scored from 0 to 4 with a higher score indicating a worse outcome, resulting in a composite score of 0-40. Functional Performance was assessed using the Australian Karnofsky Performance Scale [18]. The AKPS is an ordinal scale with 11 descriptors of function assigned a score (100 (perfect health), 90, 80, … to 0 (dead)). Data were also obtained on demographic characteristics and direct and indirect costs of care [19].

For informal carers, questionnaires assessed the role of caring (tasks in caring) and carer support needs using the 14 item carer support needs assessment tool (CSNAT) which uses a four-item descriptive (non-numerical) scale ranging from no more support needed to very much more support needed [20]. Satisfaction was assessed using the 17 item Family Satisfaction with Advanced Cancer Care (FAMCARE) scale [21]. Carer outcomes assessed included Depression and Anxiety using HADS [16], as well as Quality of Life using a single question from the EORTC QLQ C-30 which uses a rating scale from 0 to 7, where a higher score indicates better quality of life [22]. Information about carer demographics, household income and relationship to care recipient were also collected.

#### Qualitative interviews

Formal semi-structured interviews were conducted with patients and carers at Baseline and 1 month, seeking data on their experiences of living with an advanced, chronic illness, how that was treated, and what differences were noted as a result of having a case conference. We also assessed the experience of participating in this case conference and completion of patient and carer questionnaires.

In-depth interviews to identify the strengths and weaknesses of the model were held with the NP herself, and her RN colleague. Brief telephone interviews were conducted with the two senior clinicians whose organisations interfaced with the NP (Nurse Unit manager of the specialist palliative care service and the Manager of the rural district’s RACF).

### Full cohort evaluation- Health Professional practice

We described the participants and content of the case conferences that were conducted. We also audited the SCC clinical records to determine service utilization data up to 12 months prior to the case conference and 3 months after the case conference. This included hospital and emergency department visits, and NP and nurse clinical actions taken subsequent to the case conference. All health professionals involved in the CCs completed a questionnaire on the acceptability and usefulness of case conferences, and for those using telehealth, on the Jabber Video technology used for the project.

The evaluation was approved by the Human Research Ethics Committees of the University of Queensland and Queensland University of Technology. All participants provided written informed consent.

## Results

**Fig. 3:**
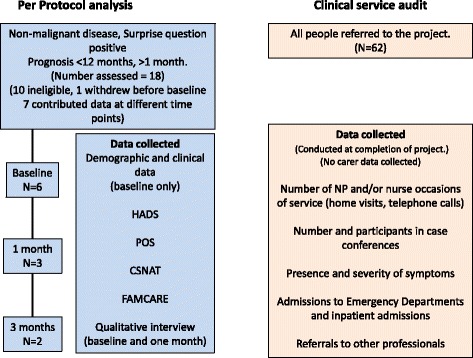
Data collection and participant flow chart

The results of the two analyses are reported separately. A description of the sample and data collection for the per protocol analysis and the cohort analysis is seen in Fig. 3.

### Patient and carer outcomes - Per protocol analysis

#### Cohort characteristics

There were 18 patients who appeared to meet initial screening criteria. Of these, ten proved ineligible. One withdrew and the remaining seven were eligible and provided data to the project. Of these six gave baseline data; 3, 1-month data and only two gave 3-month data. We present baseline data only as there were insufficient data to make meaningful interpretations of changes with time. Patient interviews from these participants will be reported elsewhere.

One of the six patients had a baseline AKPS of 70 (unable to do normal activities but cared for self); four, 50 (required considerable assistance and frequent medical care); and one, 40 (in bed >50 % of the time). The mean Palliative Outcome Scale was 27.8 (SD 8.3) and a range of 17–40. They had a mean HADS depression score of 14.7 (SD 4.7), and HADS anxiety score of score of 11.3 (SD 7.0), indicating significant depression and moderate anxiety. Five of the six had been admitted to hospital in the last 4 weeks, with a mean length of stay of 4.75 days (SD 3.0). Three had seen a specialist in the last week, three a GP and a further three had seen the GP in the last month. Five had seen a pharmacist.

Of the six carers who provided data (Table 1), three were male, four were spouses and two were adult children. All six carers lived with the index patients. Three were retired, one performed home duties and one was unemployed. One reported that they had left work to look after their partner. Most carers spent >32 h per week caring for the patient. The median hours spent performing practical tasks for the patient was 10 per week, with a range of 2–40 h per week. The median time spent performing caring tasks was 3 h, with a range of 1–40 h per week. In spite of considerable time requirements, Most carers reported remarkably few personal burdens or needs requiring external help. On average they scored normally for depression and moderately for anxiety.

**Table 1:** Carer assessment scores at baseline

Carer Support Needs Assessment tool
(Six carers, 14 potential need questions per carer; total needs considered = 84)
Potential needs with no extra help required = 60 (71%)
Potential needs with some extra help required = 22 (26%)
Potential needs with quite a bit more assistance required = 2 (2%)^a^
(One person each expressed the need for “quite a bit more assistance in “Knowing more about the patient’s illness”; and “Knowing what to expect in the future.”).
Family Satisfaction with advanced cancer care- FAMCARE-2.
Median total score 32.5, range 27–46 (Higher score, more satisfaction)
Median question score 1.9, range 1.8–7.4
Overall quality of life
Mean 4.8, SD 0.8. (Range 0–7, Higher is better)
Overall satisfaction with the care of the patient (Single item. Range: very dissatisfied to very satisfied)
5 responded satisfied or very satisfied.
HADS Depression Scale (Range 0–21)^b^
Median 6.5, range 1–9
HADS Anxiety scale (Range 0–21)^b^
Median 8.0, range 2–15

^a^Not 100 % due to rounding. ^b^ Range 0–7. low depression/anxiety; 8–10- borderline; ≥ 11- clinically significant

Qualitative data from the in-depth interviews of these patients and carers will be presented in a publication in preparation.

### Health care utilisation - Phase 2: Whole cohort assessment

#### Description of the cohort

Sixty-two patients were cared for during this pilot (Table 2). Forty were referred from the district hospital Palliative Care Service, 13 from their GP, eight from aged care facilities and one with the referral source not recorded.

**Table 2:** Patient Demographics (*n* = 62)

Female	33 (53 %)
Age	
Mean (SD) (years)	74.0 (12.3)
Median (Range) (years)	75.6 (40–97)
Recorded primary disease	
Cancer	21 (34 %)
Respiratory disease	5 (8 %)
Heart/vascular	4 (6 %)
Kidney disease	4 (6 %)
Frailty	3 (5 %)
Neurological disease	3 (5 %)
Liver disease	1 (2 %)
Dementia	0 (0 %)
No primary cause recorded	21 (43 %)

#### Patient outcomes

Twenty-seven of these patients had died at the time of the chart audit. The median time from referral to death was 11 days, with a range of 1 to 56 days. Seven of the patients who died were in the program for five days or less. We could not identify from the SCC records where patients died. There were 16 admissions from 11 patients, with a range of 1–3 admissions per patient.

1. Case conferences
  Twenty-three case conferences took place. These were conducted if the patient was not close to death at initial assessment, or if the NP considered the patient had no pressing issues at the time of assessment. Of these, three were by phone or video conferencing. In addition to the GP and NP, patients participated in two, relatives/carers in four and in one both the patient and carer participated. Other participants included the palliative care nurse (7), GP practice manager (2), GP practice nurse (2), Community or RACF nurse (7), and rural hospital Clinical Nurse Consultant (1). Table 3 shows the main symptoms that were discussed, as recorded in the SCC case notes. Pain, depression/anxiety and breathlessness were the predominant symptoms discussed.

**Table 3:** Recorded symptoms discussed in case conferences (*n* (%))

Pain (severe or moderate)	13 (25 %)
Depression and/or anxiety	12 (23 %)
Breathlessness	10 (19 %)
Nausea/vomiting	8 (15 %)
Constipation or diarrhoea	4 (7 %)
Swallowing difficulties	4 (7 %)
Urinary or bowel incontinence	2 (2 %)
Falls risk	2 (2 %)
2. Clinical actions undertaken
***Advance care planning:*** Most patients from the district hospital or from an aged care facility had an effective advance care plans (ACP) present on referral. The SCC team initiated ten new ACPs. Of these, five were referred from a hospital (out of 40 referrals), three RACFs (out of 8), and two from GPs. The remainder already had ACPs. Four patients had not appointed an Enduring Power of Attorney to manage non-health matters. They were advised to do so to avoid state intervention in their personal matters in the event of loss of competence.
3. Follow-up home visits and calls
  The nurses individually or together visited 42 of the 62 patients at home after the original assessment, with between 1 and 10 visits being made. There were 101 such visits. In addition, they made 169 phone calls to 33 patients, with a range of 1–20 calls per patient
4. Nurse practitioner initiated actions:
  The NP arranged a range of services for the patient and carer (Table 4). Most common of these were an allied health referral, arranging mobility aids or supplemental oxygen.

**Table 4:** Referral by SCC to other agencies

Allied Health (dietitian, social worker, physiotherapist, OT)	5
Mobility aids or supplemental oxygen	4
Referral to community nurse	3
Domestic help	3
Respite care	2
Arrange out-of-hours contacts	17
  The NP also prescribed 24 new medications for 12 patients, as well as altering the dose of a further nine medications in six patients (Table 5). Analgesics (including anti-epileptics and antidepressants, which can be used in neuropathic pain) were by far the most common drugs altered.

**Table 5:** Nurse practitioner initiated medication changes

Medication type	New medications (*n* = 12 patients)	Altered dose (*n* = 6 patients)
Opioids	10	3
Paracetamol	2	-
Anti-emetics	1	1
Benzodiazepines	2	-
Anti-epileptic	1	1
Antidepressant	1	-
Diuretics	1	-
Steroids	1	-
Other	5	4
Total changes	24	9
5. The model’s impact on service utilization:
  In the qualitative interviews, nurses and key stakeholders thought SMCCs avoided hospitalisation for many. No patients from an RACF went to hospital after a SMCC. Approximately 20 % of patients died at home and both nurses and all external stakeholders thought this would not have happened in most cases. They felt that the intervention helped carers know what to do in a crisis, and made it more likely that all of the resources required to manage the crisis were in place.
  Most out of hours work could be managed over the phone by the NP and RN. They found that burden was not unmanageable, and that the calls were clinically appropriate.
6. Economic viability of the service
  The interviewees thought the income generated from Medicare for NP payments was nowhere near the actual cost of the service. In particular, there is no recompense for cost of travel (and the non-billable time that was involved), the cost of time taken on telephone consultations was not covered, nor was the cost of the time of preparing for or conducting the case conferences covered. Without alternative sources of funding, they thought the service was not viable.

### Health professional experience of the model

#### Experience of case conferences

Comments from GP and other health professional participants about the process of case conferences were uniformly favorable. They felt the case conferences were efficiently and professionally run, and were very appreciative of the NP’s advice. They felt patient care was enhanced by the process, and that the carers’ needs were also being addressed.

#### The process of case conferencing

The nurses preferred face to face to phone or video conferencing, because non-verbal communication was easier to observe. They did not find video conferencing as useful for this, possibly due to poor internet speed or old equipment at the GP end.

The PEPSI COLA framework was very useful to guide and facilitate the discussion and decision-making. The nurses also used PEPSI COLA when assessing the patient. The presence of a structured assessment form allowed them to raise difficult questions with patients (“*We have to ask this question – the form tells us to!”*), allowing the collection of information otherwise very difficult to access.

Of the 23 case conferences, four involved the patient and six involved primary carers. The presence of the patient and/or carer led to filtering of the information that was being communicated. If carers were participants, the quality of the case conference was also influenced by the quality of the relationship between the patient and the carer: a strained relationship made the health professionals more guarded during the case conference.

#### Overall impression of the model

Key external stakeholders felt the service allowed them to discharge patients home with confidence that their clinical care needs would be met. It provided an excellent bridge between specialist palliative care services and the GPs and RACFs of the district. They felt that the service enabled people to remain at home where they would not otherwise have been able to. The confidence of hands-on staff to manage end of life issues improved. Relations with the ambulance service improved due to better documentation of the end of life status of the patient and the presence of advance health directives at home.

## Discussion

### Summary of findings

We have reported a project where the aim was to integrate the care of community based health professionals and particularly GPs through a NP providing expert advice and care coordination. In effect, we tested whether a NP delivering specialist advice to a primary care team for patients at the end of life was feasible and acceptable. As the NP became more involved with actual patient care than initially envisaged, the model itself evolved to one somewhat different to the “advice-only” model envisaged. However, it appears that this model may deliver benefits. This is first study to our knowledge that evaluates a non-medical specialist led model in palliative care.

Central to this process was interprofessional integration facilitated by case conferences between the patient’s service providers, and the generation of a negotiated care plan. This project demonstrated the value of a palliative care trained NPs working together with GPs in this rural setting – the first time this has been tested to our knowledge.

All sources of data from the evaluation point to improved communication and coordination of services for these patients whilst at home. Having the NP act as the single point of reference was important, particularly with respect to being available for advice. The NP also changed or initiated medicines in 18 patients, which may have stabilized at least some symptoms and reduced the need for out of hours calls or inpatient care. Case conferences led to negotiated care plans, reliable task allocation, and possibly more widespread use of more community-based resources. Patients and carers who did not have a care plan appeared to require visits to emergency departments more than those with a plan. While most patients already had ACPs at admission, the team did an extra ten.

The existence of this service allowed patients with advanced care needs to be referred home where this would previously have been problematic. The staff estimated that about 20 % of patients died at home during the project. Many more who were alive at close of the project were expected to die at home, and others to stay at home much longer than would have been possible under normal circumstances. It is also possible that the need to visit emergency departments was reduced. A larger controlled study is required to test these assumptions.

A subset of patients who were considered stable at admission to the program were not offered a case conference by the NP. These were observed to need more emergency services than those who had a case conference. This suggests that an important impact of case conferences was to provide the carers with strategies to manage predictable symptoms that had not yet emerged. We hypothesise that the negotiated care plan provided carers with confidence and the resources on hand to address the problem.

### Comparisons with other studies

In rural areas, medical practitioners are a scarce resource, and delays in access to them in the rapidly changing end of life clinical setting can be a reason for transfer to hospital. The ability to make clinical assessments and prescribe is essential, for which NPs are credentialed to do, but nurses are not. By incorporating a NP into the model, services can be more responsive to rapidly changing clinical circumstances. A systematic review of palliative care in rural areas concluded that specific interventions that involve non-medical practitioners and volunteers may improve the delivery of care [23].

There is mounting evidence that early introduction of specialist palliative care improves quality of life and survival in patients with advanced cancer [24–26]. In addition to nurses having a prominent role in these trials, nurse-led rural palliative care has also produced improvements in outcomes for advanced cancer patients [22].

Early recognition of people who are approaching the end of their life in primary care is very challenging. The low prevalence of dying in primary care settings can lead to non-recognition or late recognition of the end stages of illness. Even with tools to facilitate recognition of these people, only a fraction of them are actually identified in a timely manner at present [27–29].

Once patients are identified, there is an opportunity for early intervention and this is the place of models such as this one. Cooperation between medical and nursing care providers must occur to occur to produce acceptable care. Randomised trials involving SMCCs between palliative care specialists and the patient’s GP in predominantly cancer care have produced important improvements [9, 10]. Pilot studies in patients with non-malignant organ failure identified in hospitals, and deliberate outreach to their GPs through case conferencing appears to confer very significant benefits to this group of patients [7].

The care reported in this paper combines nurse led care and GP-specialist NP cooperation using SMCCs. Ideally these initiatives occur early in the course of the person’s final illness. This pilot study suggests that a NP-led integrated model of care may provide improvements in care in a similar manner to palliative medicine specialists providing input into SMCCs.

### Lessons learned

The NP’s initial assessments unearthed urgent clinical issues in advanced palliative patients who were not the original target of the project, highlighting the need for earlier identification of patients who may benefit from specialist input. The follow-up work that flowed from these advanced palliative patients, including after-hours commitments, was resource intensive.

Early in the study, the nurses elected not to arrange case conferences for those people they thought were stable and not requiring clinical decisions to be made. On reflection, they started to see that those patients were the ones being readmitted to hospital, because their families did not know what to do and were not prepared for predictable exacerbations. They rectified this later in the study. This highlights the strength of the model – that planning before complications arise can lead to prompt, effective treatment at home. Resources for the exacerbations are in place and phone advice is available. Patients and their carers are empowered to manage anticipated, with hospital as a backup if the plans do not work.

The NP and her senior nurse shared out-of-hours calls, and visited the patient if the problems warranted it. This service was not part of the project brief, and reflected their commitment to their patients. While there were few out-of-hours phone calls and fewer home visits, future service models need to ensure reliable out-of-hours service provision. The model was clearly dependent on the commitment of the participants, and raises issues relating to professional boundaries and the need for care protocols to guide practitioners in these settings. Better out-of-hours arrangements or a higher workload would require extra staff to share the on call burden.

One major expense was the cost of travel to clients in rural areas from the regional centre. Neither travel time nor transport costs were met through the Medicare rebate for NPs. Clearly the funding model assumes that NP work occurs in one place, which limits its effectiveness for rural NPs. The model we tested is not adequately supported by current funding systems. Without supplementary program funding, gaps in services would recur.

Telehealth is an option that could overcome this problem. Telehealth is being used for home reviews of patients, as well as support and advice to patients, carers and clinicians, and acceptable for all parties [30]. It may be cost effective in rural care. For example, a paediatric palliative care telehealth program in Queensland, Australia, and showed cost savings of approximately $AU1000 per specialist home consultation [31].

From a whole of system perspective, and in light of previous research into case conferences in non-malignant disease [6], the model trialed in this project has the potential to produce net savings through hospital avoidance. This arises through better inter-professional communication, generating more confidence in patients and particularly their carers, and improved ability to manage symptoms. However, the system is fragmented, and costs incurred in one part of the system do not get recompensed for savings experienced in another part [7, 8].

### Strengths and weaknesses

This project was conceived with a patient population with non-malignant, end stage conditions most commonly found in community care in mind, and the introduction of patients with far more advanced illness (usually cancer) than was planned for led to major deviations from the original care model. This rendered the initial evaluation plan unworkable for many patients. We thus conducted a two-phase evaluation. There was no control group, so the descriptive data presented can only lead to inferences of any effect. Data collection had to fit with the realities of clinical practice. As a result, there were few per-protocol patients, some missing data, and not all desired data were available from the SCC clinical records available to the evaluators. However, the larger dataset improved the face validity of the evaluation, as did the mixed methods technique used.

## Conclusion

A NP-coordinated care model which includes formal case conferences with local GPs may improve coordination of care, and patient and carer confidence in managing symptoms and may make dying home in a rural setting more feasible. The funding model requires sufficient support to account for the extra costs of travel required to visit patients at home. Telehealth patient reviews and case conferences may make this model more economically feasible.
